# Cardiorespiratory Fitness and Its Place in Medicine

**DOI:** 10.31083/j.rcm2401014

**Published:** 2023-01-06

**Authors:** Robert Ross, Jonathan Myers

**Affiliations:** ^1^School of Kinesiology and Health Studies, Department of Medicine, Division of Endocrinology and Metabolism, Queen’s University, Kingston, Ontario K7P 3E8, Canada; ^2^School of Medicine, VA Palo Alto Health Care System, Stanford University, Palo Alto, CA 94304, USA

**Keywords:** cardiorespiratory fitness, morbidity, mortality, physical activity, exercise, high intensity interval training

## Abstract

The evidence that cardiorespiratory fitness (CRF) predicts morbidity and mortality independent of commonly obtained risk factors is beyond dispute. Observations establishing that the addition of CRF to algorithms for estimating cardiovascular disease risk reinforces the clinical utility of CRF. Evidence suggesting that non-exercise estimations of CRF are associated with all-cause mortality provides an opportunity to obtain estimates of CRF in a cost-effective manner. Together with the observation that CRF is substantially improved in response to exercise consistent with guideline recommendations underscores the position that CRF should be included as a routine measure across all health care settings. Here we provide a brief overview of the evidence in support of this position.

## 1. Introduction

Over the past few decades several reviews and commentaries have been published wherein the authors conclude that the time has come for cardiorespiratory fitness (CRF) to be a vital sign across health care settings and in particular, primary care [1]. This is not surprising as there is now undisputed evidence to confirm that CRF is associated with morbidity and mortality independent of commonly obtained risk factors [1, 2, 3, 4, 5, 6, 7, 8] and improves risk stratification [1]. It is also established that estimates of CRF can be obtained in a pragmatic manner and, that CRF can be improved in response to exercise that is consistent with current recommendations [9, 10]. Here we update the evidence that supports the recommendation that the routine incorporation of CRF into health care settings reflects best evidence and consequently, will improve patient/client management.

## 2. CRF Independently Predicts Morbidity and Mortality

There is now indisputable evidence that establishes a negative, inverse relationship dose-response relationship between CRF, morbidity and mortality. Previous meta-analyses establishing a dose-response relationship between CRF and mortality [2, 3] were recently confirmed by Laukkanen *et al*. [5]. The authors’ meta-analysis included data from 37 studies comprising over 2 million adults with objective measures of CRF. In this study the authors reported that the relative risk for all-cause mortality was reduced by 11% for every 1-metabolic equivalent (MET) increase in CRF independent of age, biological sex, and duration of follow-up. The authors also observed a risk reduction of 45% among adults in the highest tertile compared to those in the lowest tertile: a finding consistent with the frequently reported observation that the mortality benefit is best among adults who move from the least fit to the next fit group [3, 6, 7, 8].

Early findings that established an association between CRF and health outcomes were based on a single measurement of CRF obtained at baseline. Blair and colleagues [11] were among the first to demonstrate an association between changes in CRF and mortality. A principal observation was that in men who maintained CRF (fit) over 5 years, the relative risk for all-cause mortality and cardiovascular disease (CVD) was reduced by 67% and 78% respectively, in comparison to men who remained unfit. This observation remained true independent of commonly obtained risk factors. This seminal observation has repeatedly been confirmed [1, 12].

## 3. CRF Improves Risk Estimates for Morbidity and Mortality

A growing body of evidence now indicates that adding CRF to risk engines designed to calculate the absolute risk for CVD (e.g., the Framingham coronary heart disease (CHD) risk assessment algorithm) enhances risk stratification [1]. Net reclassification improvement (NRI) is a statistical approach that has recently been used to determine the degree to which a selected biomarker adds to existing markers to predict health outcomes. Indeed, when compared to the risk calculated using traditional risk factors (e.g., age, biological sex, hypertension, diabetes, hyperlipidemia, and smoking), the addition of CRF to traditional risk factors results in positive NRI values in the range of 10 to 30% [13, 14, 15, 16, 17]. These observations clearly demonstrate that the addition of CRF to traditional models substantively improves the ability to estimate risk for all-cause mortality and cardiovascular events. It is also encouraging that these observations remain whether CRF was measured objectively or estimated using non-exercise algorithms.

That the addition of CRF to traditional risk factors results in significant improvement in risk prediction is clinically relevant and addresses a major concern raised by those who remain unconvinced that CRF should be a vital sign routinely measured in clinical settings. In short, CRF remains a simple evidence-based target within all clinical settings and provides practitioners with an opportunity to counsel patients/clients on the health benefits of lifestyle-based strategies designed to reduce health risk. Thus, in addition to improving risk prediction modeling, CRF serves as an important modifiable treatment target for risk reduction.

## 4. Non-Exercise Estimates of CRF

In order for CRF to gain traction as a risk factor considered of equal importance to traditional risk factors and to be routinely applied in clinical practice, it needs to be simple, rapid and inexpensive to obtain. While the most accurate metric for CRF requires a maximal exercise test, it is neither feasible nor appropriate to perform an exercise test during routine clinical encounters. In addition to time and cost factors, performing an exercise test in most individuals does not meet appropriate use criteria [18]. A 2018 update of the US Preventive Services Task Force (USPSTF) Recommendations on Resting or Exercise Electrocardiography [19] did not recommend routine exercise testing for asymptomatic individuals. This is in accordance with earlier recommendations from the USPSTF [20], and other guidelines on exercise testing [21, 22]. This recommendation is based in part on the limited predictive accuracy of the test (the percentage of times the test provides a correct result) in asymptomatic individuals and its low cost-effectiveness. These guidelines are consistent in recommending that an exercise test should generally be performed only in patients with known or suspected CVD.

Because an exercise test cannot be conducted routinely in most individuals, there has been growing interest in the use of non-exercise methods to estimate CRF. These studies have incorporated demographic and risk factor information that is easily available at the time of a clinic visit such as age, body mass index, symptom questionnaires, physical activity patterns, smoking history, and other factors that have a potential impact on CRF. A synopsis of key studies that have developed multivariable models to estimate CRF from non-exercise data is shown in Table 1 (Ref. [23, 24, 25, 26, 27, 28, 29, 30, 31, 32, 33, 34, 35, 36, 37, 38, 39]). Several observations are notable from the table. First, the associations between estimated and objectively measured CRF (CRF determined by indirect calorimetry or estimated from peak work rate) range in the order of 0.60 to 0.85 (using the coefficient of determination, or $R^{2}$). This degree of association appears to be generally adequate in terms of classifying individuals into CRF categories (e.g., quartiles or quintiles). In real terms, the error between estimated and measured CRF is generally in the range of 5–15% [23, 24, 25, 31, 35]. Nes *et al*. [35] for example, studied >4000 men and women using a non-exercise test model to estimate CRF and reported that >90% of subjects were correctly classified into the lowest and highest quartiles of CRF. The available equations have tended to underestimate CRF among higher fit individuals and overestimate CRF among lower fit individuals [23, 24, 26, 28, 31, 35]. This is generally not an issue among highly fit individuals who would still be correctly classified into the higher CRF categories but is a potential concern for low fit individuals because correct classification is much more likely to influence their estimation of risk. Variation in results of the studies can be attributed to differences in the populations studied, the fact that accessible non-exercise variables differed in the different samples, and differences in the methods of expressing the association between estimated and measured exercise capacity. Generally speaking, the error and variation in estimated CRF is similar to that for day-to-day variation in other risk factors such as blood pressure or lipids [40, 41]. There are several clinical situations in which the measurement of CRF requires precision and therefore a maximal exercise test, but this degree of variation suggests that the available non-exercise estimates are acceptable for the purposes of applying CRF as a risk factor, for physical activity counseling, or for many research purposes.

**Table 1.** **Selected non-exercise equations to estimate cardiorespiratory fitness**.

Authors	Population	Gender	n	Age	Equation	$R^{2}$	SEE
Jackson *et al*. (1990) [23]	Employees of NASA	M/F	1393/150	20–70	50.513 + 1.589 (PAR 0–7) – 0.289 (age in years) + 5.863 (sex, male = 1 and female = 0) – 0.552 (%fat)	0.66	5.35
Myers (1994) [24]	Veterans referred for an exercise test	M	212	62 ± 8	4.7 + 0.97 (VSAQ) – 0.06 (age)	0.67	1.43
Heil *et al*. (1995) [25]	Healthy	M/F	210/229	20–79	36.580 + 1.347 (activity 0–7) + 0.558 (age in year) – 0.00781 (${\mathrm{age}}^{2}$) + 3.706 (sex, male = 1 and female = 0) – 0.541 (%fat)	0.77	4.90
Whaley *et al*. (1995) [26]	Active adults	M/F	702/473	41.8 ± 11	61.66 + 1.832 (PAS 1–6) – 0.328 (age in year) + 5.45 (sex, male = 1 and female = 0) – 0.446 (smoking 1–8) – 0.436 (%fat) – 0.143 (RHR)	0.73	5.38
George *et al*. (1997) [27]	Active college students	M/F	50/50	18–29	44.895 + 0.688 (PAR 0–10) + 7.042 (sex, male = 1 and female = 0) – 0.823 (self-reported BMI) + 0.738 (PFA 1–3)	0.71	3.60
Matthews *et al*. (1999) [28]	Healthy	M/F	390/409	19–79	34.142 + 1.463 (PAS 0–7) + 0.133 (age in year) – 0.005 (${\mathrm{age}}^{2}$) + 11.403 (sex, male = 1 and female = 0) – 0.254 (WT in kg) + 9.170 (HT in m)	0.74	5.64
Malek *et al*. (2004) [29]	Aerobically trained	F	80	38 ± 9.5	22.931 + 0.392 (h/wk training) + 1.035 (RPE 6–20) + 4.368 (natural log of years of training) – 0.287 (age in year) + 0.309 (WT in kg) + 0.200 (HT in cm)	0.67	4.32
Malek *et al*. (2005) [30]	Aerobically trained	M	112	40.2 ± 11.7	57.912 + 0.329 (h/wk training) + 1.444 (RPE 6–20) + 6.366 (natural log of years of training) – 0.346 (age in year) + 0.344 (WT in kg) + 0.335 (HT in cm)	0.65	4.75
Jurca *et al*. (2005) [31]	ACLS	M/F	35,826/10,364	20–70	65.835 + 2.838 (activity1) + 4.095 (activity2) + 7.56 (activity3) + 10.675 (activity4) – 0.28 (age in year) + 8.715 (sex, male = 1 and female = 0) – 0.595 (BMI) – 0.175 (RHR)	0.60	5.25
Bradshaw *et al*. (2005) [32]	Healthy	M/F	50/50	18–65	48.073 + 0.671 (PAR 0–10) – 0.246 (age in year) + 6.178 (sex, male = 1 and female = 0) – 0.619 (BMI) + 0.712 (PFA 1–13)	0.86	3.44
Cao *et al*. (2010) [33]	Healthy	F	148	20–69	51.853 + 0.408 (SC, ${\mathrm{10}}^{3}$ steps/day) + 0.060 (MVPA in min) – 0.175 (age in year) – 0.244 (WC in cm)	0.72	3.14
Cao *et al*. (2010) [34]	Healthy	M	127	20–69	61.925 + 0.577 (SC, ${\mathrm{10}}^{3}$ steps/day) + 0.305 (VPA in min) – 0.338 (age in year) – 0.698 (BMI)	0.71	4.15
Nes *et al*. (2011) [35]	Healthy	M/F	2067/2193	48.4 ± 13.6	100.27 + 0.226 (PA index 0–8.3) – 0.296 (age) – 0.369 (WC in cm) – 0.155 (RHR) for men	0.61	5.70
					74.74 + 0.198 (PA index 0–8.3) – 0.247 (age) – 0.259 (WC in cm) – 0.114 (RHR) for women	0.56	5.14
Jang *et al* (2012) [36]	Healthy	M/F	113/104	34.2 ± 8.4	43.98 – 0.12 × age + 11.64 × gender (0 = female; 1 = male) – 0.271 × BMI – 1.36 × Smoking (0 = never or quit; 1 = current) + 0.70 × LTPA + 1.05 × ATC + 0.03 × ATD + 0.035 × BMR + 0.72 × heavy physical work	0.79	3.36
Maranhão Neto *et al*. (2012) [37]	Cardiovascular/metabolic disease	M/F	109	69.1 ± 7.4	6.095 *– *0.096 (Age) + 8.84 (Handgrip	0.79	1.1 (METs)
					strength/WT) + 0.67 (RPC)		
Sloan *et al* (2022) [38]	Healthy	M/F	42,676	44.1 ± 9.6		0.70 (men)	1.7
						0.65 (women)	1.6
Myers *et al*. (2022) [39]	Veterans referred for an exercise test	93% M	1545	60 ± 13	5.1 + (0.67 × VSAQ) – (0.09 × BMI) – (0.59 × Smoking) – (1.2 × CHF) – (0.46 × β-blocker) – (0.45 × HTN) + (0.45 × CAD) + (0.49 × DOE) + (1.1 × CP)	0.67	

SEE, standard error of estimate (in mL/kg/min); PAR, physical activity rating; VSAQ, Veterans Specific Activity Questionnaire; PFA, perceived functional ability; PAS, physical activity status; WT, weight; HT, height; BMI, body mass index; RHR, resting heart rate; NASA, National Aeronautics and Space Administration; ACLS, Aerobics Center Longitudinal Study; WC, waist circumference; RPE, rate of perceived exertion; MVPA, moderate-to-vigorous physical activity; VPA, vigorous physical activity; LTPA, leisure time physical activity; ATC, ambulation time during commute; ATD, ambulation time on duty; BMR, body motion rate; RPC, rating of perceived capacity; CHF, chronic heart failure; HTN, hypertension; CAD, coronary artery disease; DOE, dyspnea on exertion; CP, chest pain.

There are a number of notable differences between the various non-exercise methods to estimate CRF. Approaches to estimating CRF have ranged from submaximal cycle or treadmill tests, walking tests, field tests, and the application of clinical and demographic data that is readily available from clinical records or questionnaires at the time of an encounter. Many early studies in this area relied on field tests, and while these studies reported reasonable associations with measured peak ${\mathrm{VO}}_{2}$ (the highest value of ${\mathrm{VO}}_{2}$ attained during an incremental exercise test) from an exercise test [42, 43, 44, 45, 46, 47, 48, 49], they are impractical to apply in large populations or as widely used public health tools. Moreover, field, or submaximal tests are generally not more accurate than the use of non-exercise data available at the time of an encounter [1, 42, 43, 44, 45, 46, 47, 48, 49]. The most appropriate method to estimate CRF from non-exercise data will undoubtedly differ depending upon the context in which CRF is applied and the sample being studied. For example, applying a symptom questionnaire (such as the Veterans Specific Activity Questionnaire [24, 39] or Duke Activity Status Index [50]) are suitable for clinically referred samples (the group for which they were developed), but most of the models have been derived from relatively healthy, asymptomatic subjects for whom these tools would not apply. Not all samples had physical activity patterns available, which is the key behavioral factor influencing CRF. Indeed, in many studies, physical activity patterns explained a significant proportion of variance in exercise capacity [23, 31, 32, 33, 34, 35, 51]. The addition of variables such as gender, age, height, weight, and/or BMI to models has generally improved the accuracy of the equations; these variables are particularly appropriate when there is significant variation in the population characteristics. In clinical settings, an optimal approach might be to automatically provide estimations of CRF as part of electronic medical records so that they are available at the time of a clinical encounter, as has been advocated for physical activity behavior [52].

## 5. Role of Non-Exercise CRF in Epidemiologic Studies

A rapid and reasonably accurate non-exercise estimate of CRF would be particularly useful when testing large populations or performing epidemiologic research, in which exercise testing of large numbers of participants is impractical. A growing number of studies have applied estimates of CRF derived from a non-exercise prediction model to estimate future risk of mortality, CVD events or cancer [15, 39, 53, 54, 55]. Notably, the risk reductions per each 1-MET higher non-exercise estimate of CRF have been demonstrated to be similar to those using measured exercise capacity from a treadmill or cycle ergometer (10–20%). Among 43,356 subjects from the Aerobics Center Longitudinal Study, CRF was estimated using sex, body mass index, age, waist circumference, physical activity level, resting heart rate and smoking status [55]. After adjustment for potential confounders, both estimated and measured CRF were inversely associated with non-fatal CVD events, CVD mortality and all-cause mortality in men, and with non-fatal CVD and all-cause mortality in women. Importantly, measured CRF had superior discriminative ability than estimated CRF (c-statistic 0.70 vs. 0.64 for all-cause mortality and 0.74 vs. 0.73 for CVD mortality). Using similar non-exercise test variables, Stamatakis *et al*. [15] followed 32,319 subjects for a mean of 9 years and observed that a higher non-exercise CRF score was associated with a lower risk of mortality from all-causes (hazard ratios per SD increase; 0.85 in men and 0.88 in women) and CVD (hazard ratios 0.75 in men and 0.73 in women). Both of these studies reported that the discriminative utility of estimated CRF was higher than that from any of its individual components, separately or together, for all-cause mortality and CVD events. In fact, by adding non-exercise CRF, Stamatakis *et al*. [15] reported NRI for CVD mortality (compared to a standardized aggregate score of modifiable risk factors) of 27.2% and 21.0% for men and women, respectively. Thus, for large population-based observational studies, non-exercise estimates generally appear to provide adequate reflections of CRF, although they are somewhat less powerful than directly measured CRF. Nevertheless, these and other studies applying non-exercise estimates of CRF [15, 23, 24, 25, 26, 27, 28, 29, 30, 31, 32, 33, 34, 35, 36, 37, 38, 39, 56] provide further confirmation of the power of CRF in predicting risk for adverse outcomes.

## 6. CRF Response to Physical Activity

**Fig. 1.**
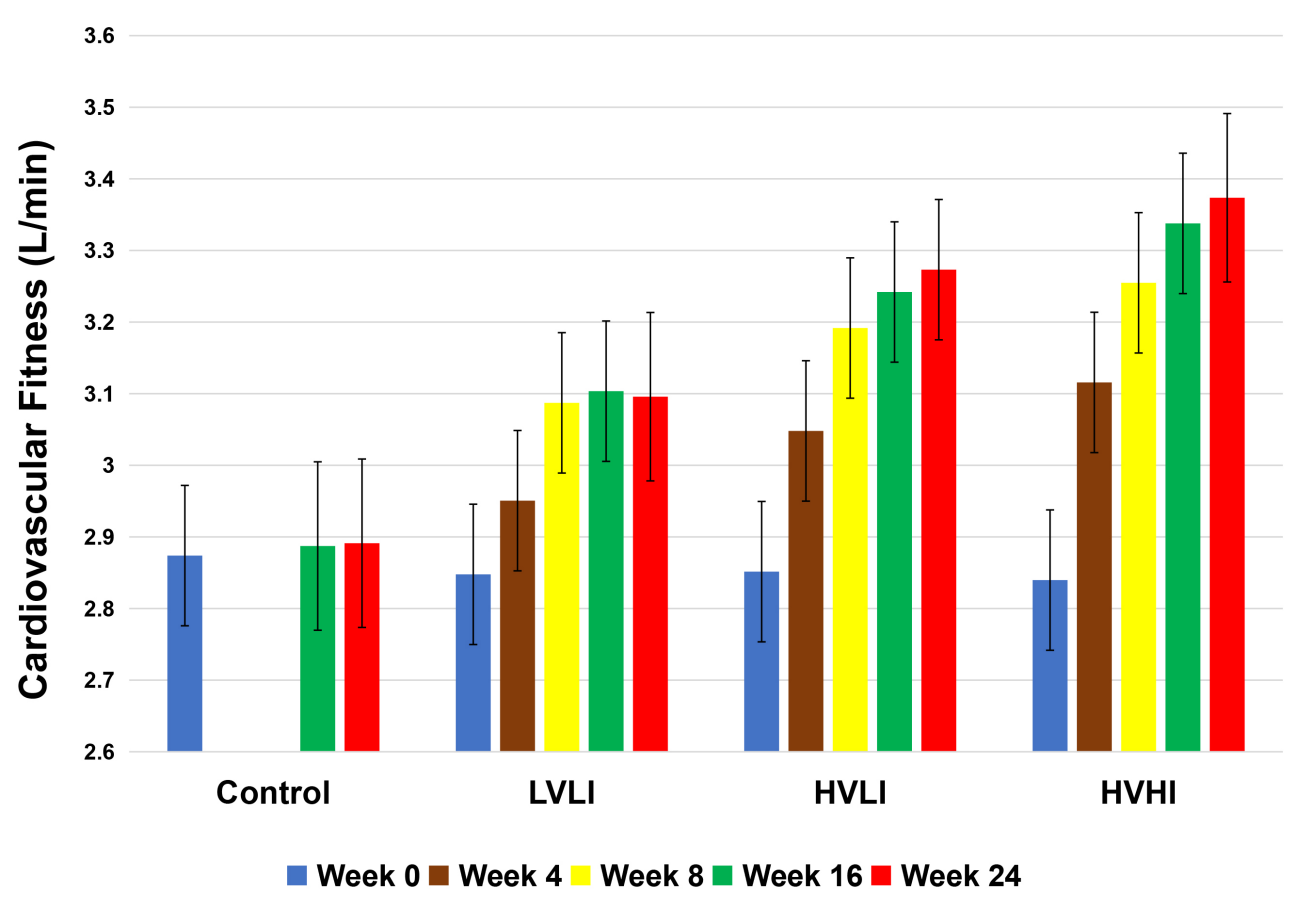
**Association between exercise dose and change in ${\mathrm{VO}}_{2}$peak over 24 weeks**. Figure adapted from Ref. [10]. Values represent the observed change in ${\mathrm{VO}}_{2}$peak at 4, 8, 16 and 24 weeks in adults with a mean age of 53 years. ${\mathrm{VO}}_{2}$peak was not measured at weeks 4 and 8 within the control group. Values are least-squares estimated means adjusted for age and sex. HAHI, high amount high intensity; HALI, high amount low intensity; LALI, low amount low intensity (see Ref. [10] for details). At baseline, there were no differences between groups. At weeks 4, 8, 16, and 24, the increase in ${\mathrm{VO}}_{2}$peak was greater for the HAHI group than the LALI (*p *< 0.001) and control (*p *< 0.001) groups. At weeks 16 and 24, the increase in ${\mathrm{VO}}_{2}$peak was greater for the HALI group than the control (*p *< 0.001) and LALI (*p *< 0.001) groups. The increase in ${\mathrm{VO}}_{2}$peak for the HAHI group was greater than the HALI group at 8, 16, and 24 weeks (*p* = 0.03, 0.002, and 0.03, respectively).

Although heritability accounts for about 50% of the individual variation in the response of CRF to exercise [57], it is firmly established that for most adults, CRF increases in response to regular physical activity (PA). To achieve PA-induced health benefits, the consensus recommendation worldwide calls for adults to accumulate about 150 minutes of moderate-to-vigorous PA weekly [58, 59, 60]. The findings from numerous, rigorously controlled randomized trials confirm that exercise performed at levels consistent with current recommendations is associated with improvements in CRF regardless of age or biological sex [7, 9, 10, 61]. Several trials have also considered the interaction between exercise amount (determined by exercise minutes or kilocalories expended) and exercise intensity on the CRF response [10, 61]. Our prior review of these trials concluded that while increasing exercise amount or intensity is associated with positive, dose-response increases in CRF, exercise intensity appeared to be the strongest driver of the increase in CRF [62]. The interaction between exercise amount and intensity is nicely illustrated in the findings presented in Fig. 1. In that study adults with abdominal obesity were randomized to 1 of 3 groups that varied in exercise intensity and amount. The results confirm that, when exercise is fixed at an intensity approximating 50% of ${\mathrm{VO}}_{2}$peak, CRF increases with increasing exercise amount, a finding consistent with others [62]. Furthermore, for a fixed amount of exercise, CRF also increases with increasing exercise intensity.

## 7. High Intensity Interval Training and CRF 

Whether high intensity interval training (HIIT) is associated with improvements in CRF that are greater compared to moderate intensity continuous training (MICT) has also been the subject of increasing interest. A meta-analysis was performed by Gist and colleagues [63] to determine the effects of high intensity (exercise performed at greater than 100% of ${\mathrm{VO}}_{2}$peak) sprint interval training (SIT) on CRF. The authors assessed the findings from 16 randomized controlled studies that included 318 adults with an average age of 24 years who performed SIT exercise for about 5 weeks. The primary finding was that SIT-induced a statistically greater improvement ${\mathrm{VO}}_{2}$peak (3.6 mL/kg/min or 8%) relative to no-exercise controls. Interestingly, however, the SIT-induced improvement in ${\mathrm{VO}}_{2}$peak was not different when compared to MICT in which exercise was performed continuously at about 65% of ${\mathrm{VO}}_{2}$peak.

The finding that SIT-induced improvement in CRF was not different from those observed in response to MICT differs from the observations of Sultana *et al*. [64] who performed a systematic review to determine the effect of HIIT versus a non-exercising control and MICT on CRF in adults with normal weight, overweight and obesity. In this analysis MICT was defined as aerobic exercise performed continuously at steady state for a duration approximating 20–60 minutes at a moderate intensity (40% to 60% of ${\mathrm{VO}}_{2}$peak or heart rate reserve). MICT was compared to both HIIT (intervals performed at ~85% ${\mathrm{VO}}_{2}$peak) and SIT (>than 100% of ${\mathrm{VO}}_{2}$peak) interventions. The findings based on a review of 47 studies revealed a significant difference between HIIT and non-exercising controls, favoring HIIT (effect size (ES): –0.788, 95% confidence interval (CI) –0.957 to –0.620; *p *< 0.001), and between HIIT and MICT, favoring HIIT (ES: –0.175, 95% CI –0.318 to –0.031; *p* = 0.017).

Finally, a systematic review was performed by Weston and colleagues [65] to determine the utility and safety of HIIT in persons with cardiometabolic disease. The authors retrieved 10 studies with 273 patients heart disease, hypertension, metabolic syndrome, and obesity. The principal observation was that CRF was increased to a 9% greater extent in response to HIIT compared to MICT (mean difference: 3.03 mL/kg/ min, 95% CI 2.00 to 4.07). The observed increase in HIIT was almost double that of MICT in patients with lifestyle-induced chronic diseases.

Thus, the weighted evidence supports the observation that substantial improvements in CRF are observed in response to physical activity/exercise consistent with consensus recommendations. It also appears that exercise intensity, either in response to continuous exercise or HIIT, drives improvement in CRF. Whether HIIT has far-reaching public health implications remains to be determined. Most of the HIIT studies are of short duration and thus, whether participation would be sustained for long durations is unknown. Also unclear is whether most adults would have routine access to a stationary cycle or a treadmill that may be required to perform interval training safely. These unanswered questions are important, and answers are required before one concludes with confidence that HIIT is a feasible option for improving CRF.

## 8. Conclusions

CRF provides information to health care providers that improves patient management independent of age, sex, and race/ethnicity. Undisputed evidence has established that CRF is inversely associated with morbidity and mortality independent of commonly obtained risk factors, improves risk stratification, can be obtained in a pragmatic manner, and is substantively improved in response to exercise consistent with current recommendations. That its’ assessment provides additional opportunities to counsel patients on the benefits of physical activity serves to reinforce the recommendation that CRF be a routine measure in all health care settings.
